# Recurrent ovarian cancer presenting in the right supraclavicular lymph node with isolated metastasis: a case report

**DOI:** 10.1186/1752-1947-6-176

**Published:** 2012-07-02

**Authors:** Tomohito Tanaka, Masahide Ohmichi

**Affiliations:** 1 Department of Obstetrics and Gynecology, Osaka Minami Medical Center, 2-1, Kidohigashi-machi, Kawachinagano, Osaka, 586-8521, Japan; 2 Department of Obstetrics and Gynecology, Osaka Medical College, 2-7, Daigaku-machi, Takatsuki, Osaka, 569-8686, Japan

## Abstract

**Introduction:**

The majority of ovarian cancer recurrences are in the abdomen. However, some cases relapse as isolated lymph node metastases, mostly in pelvic or para-aortic nodes. Peripheral isolated lymph node metastasis is rare.

**Case presentation:**

A 69-year-old Japanese woman had recurrent ovarian cancer presenting with isolated right supraclavicular lymph node metastasis. After surgical resection and combination chemotherapy with carboplatin and paclitaxel, her right supraclavicular lymph node completely regressed.

**Conclusions:**

Peripheral isolated lymph nodes, including right supraclavicular lymph node, can recur without a macroscopic abdominal lesion. Clinicians should carefully examine peripheral lymph nodes for recurrence.

## Introduction

The majority of ovarian cancer (OC) recurrences are within the abdomen. However, some cases relapse as isolated lymph node metastases, mostly in pelvic or para-aortic nodes; peripheral isolated lymph node metastasis is rare. Left supraclavicular lymph node (LSCLN), better known as Virchow’s node, collects lymph through the thoracic duct and from most areas of the body (mainly the abdomen). A finding of an enlarged node has been regarded as strongly indicative of the presence of cancer in the abdomen. In contrast, right supraclavicular lymph node (RSCLN) takes its supply mainly from the mediastinum, lungs, and esophagus [1]. We report a case of recurrent OC presenting with an isolated RSCLN metastasis with no evidence of any other recurrent part after 52 months from initial surgery.

## Case presentation

**Figure 1:**
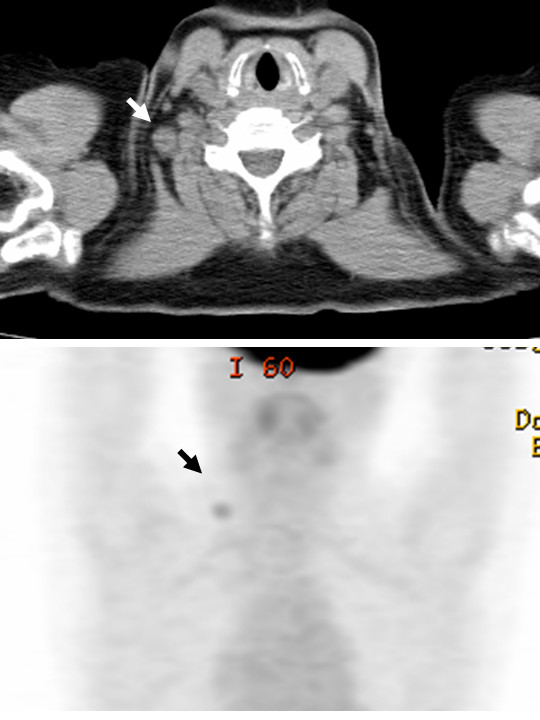
Fluorine-18 fluorodeoxyglucose positron emission tomography associated with computed tomography images revealed an area of increased metabolic activity only in the right supraclavicular lymph node (arrows).

A 65-year-old post-menopausal Japanese woman (gravida 2, para 2) with abdominal distension had an ovarian tumor that was 15 cm in diameter. She had a medical history of vaginal hysterectomy because of a uterine prolapse after menopause at the age of 55 years. Her levels of serum cancer-related antigen 125 (CA125) and carbohydrate antigen 19-9 (CA19-9) were 170.0U/mL (normal range is 0 to 35U/mL) and 15.1U/mL (normal range is 0 to 37U/mL), respectively. She underwent a bilateral salpingo-oophorectomy, an omentectomy, and a pelvic lymphadenectomy. The surgery revealed that the tumor originated from her bilateral ovaries. No evidence of pelvic spread, peritoneal implantation, or ascites was found. The results of a peritoneal washing cytological examination were negative. On palpation, the para-aortic lymph nodes were found not to be swollen. The final diagnosis was stage Ib serous adenocarcinoma. The serum concentration of CA125 decreased to below the normal limit after surgical removal of the tumor. Subsequently, our patient underwent six cycles of paclitaxel (180 mg/m^2^; three-hour intravenous infusion) given on day one plus carboplatin of the area under the curve (AUC) of 5 mg given on day one of a 21-day cycle (TC). Follow-up abdominal and pelvic computed tomography (CT) at the end of chemotherapy showed no evidence of recurrent or residual lesions in the pelvis. After 52 months from the initial surgery, our patient found a firm mass in her right supraclavicular area. There was no evidence of other lymphadenopathy, ascites, or abdominal masses. The results of a breast examination were normal, the thyroid gland was not palpable, and there were no skin lesions. The serum CA125 level increased to 69.6U/mL. CT demonstrated RSCLN swollen to 15 mm in diameter. Other examinations, such as mammography, chest and abdominal CT, gastroscopy, colonoscopy, and gallium-67 scintigraphy, revealed no abnormal findings. A core needle biopsy showed adenocarcinoma, similar to the findings from the primary OC: our patient had a clinical diagnosis of isolated RSCLN metastasis from OC. On palpation, the mass was immobile and adhered well to a wide vessel surface. Surgical resection might have placed our patient at risk of peri-operative mortality. Our patient then underwent neoadjuvant chemotherapy of dose-dense paclitaxel (80 mg/m^2^; one-hour intravenous infusion) given on days one, eight, and 15 plus carboplatin in the AUC of 5 mg given on day one of a 21-day cycle (wTC). After three cycles of wTC, the serum CA125 level decreased to the normal range and the relapse lesion could not be palpated. The relapse lesion was also undetected on CT and was completely regressed. Our patient was then given another three cycles of wTC without surgical resection. However, 17 months after the relapse was noticed, our patient found the RSCLN growing again. Fluorine-18 fluorodeoxyglucose positron emission tomography associated with computed tomography (PET/CT) images revealed an area of increased metabolic activity only in the RSCLN (Figure 1). Our patient underwent surgical resection because the relapse lesion was highly mobile and seemed to have no adhesion. After the surgical resection, four cycles of wTC were administered. Our patient was free of disease at a 20-month follow-up consultation after the resection of RSCLN.

**Figure 2:**
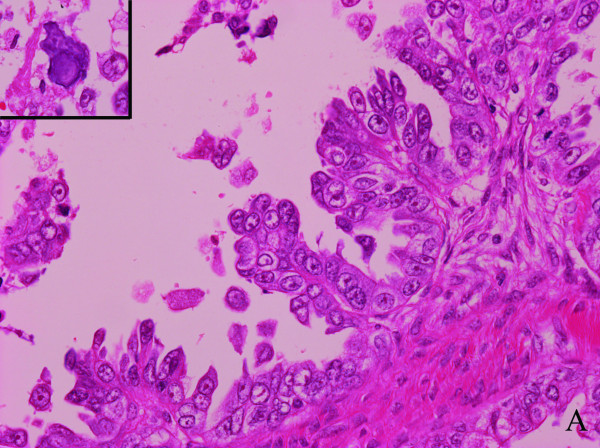
**Histological findings of the tumor. (a)** The primary ovarian tumor was composed of closely packed irregular papillae, some of which had fibrous cores, lined by cells with stromal invasion. The tumor cells had atypical nuclei with prominent nucleoli and high nuclear-to-cytoplasmic ratios. Psammoma bodies were occasionally seen (upper left). **(b)** A specimen from right supraclavicular lymph node composed of cuboidal cells with oval to round nuclei with papillary infiltration. Psammoma bodies were frequently seen. (a,b) Stain: hematoxylin and eosin; magnification: ×400.

At the initial surgery, the diameters of the right and left ovarian tumors were 120 × 78 × 60 mm and 80 × 68 × 60 mm, respectively. The tumors had smooth lobulated external surfaces. The cut surfaces revealed multiple papillary solid nodules within a thick-walled unilocular cyst containing yellowish serous fluid. A microscopic examination showed that the tumors were composed of closely packed irregular papillae, most of which had fibrous cores, lined with cells with stromal invasion. The tumor cells had atypical nuclei with prominent nucleoli and high nuclear-to-cytoplasmic ratios. Psammoma bodies were occasionally seen (Figure 2a). Immunohistochemically, the tumor cells were positive for cytokeratin 7 (CK7) and CA125 and negative for CK20 and CA19-9. The staining for Wilms’ tumor 1 protein (WT1) was positive in the nuclei of the cancer cells.

In contrast, a specimen from RSCLN showed findings similar to those in the ovaries. The tumor cells, composed of cuboidal cells with oval to round nuclei, had papillary infiltration. Psammoma bodies were frequently seen (Figure 2b). Immunohistochemically, the tumor cells were positive for CK7, CA125, and WT1 and negative for CK20 and CA19-9.

## Discussion

The most common routes for the spread of epithelial OC are lymphatic dissemination and transcoelomic spread through the nearby internal organs [2]. Although isolated lymph node recurrence (ILNR) of OC is relatively rare, the literature includes several reports of large numbers of ILNRs [3-9]. In these reports, ILNR occurred in about 4% to 6% [3,4] of patients with OC, representing approximately 10% [3] of the overall recurrences. In contrast, the clinical presentation of extra-abdominal lymphadenopathy without abdominal mass can occur before evidence of ovarian mass [10]. In 205 patients with ILNR of OC, although the most frequently involved sites were para-aortic or pelvic or both, other sites, such as left supraclavicular (eight cases), inguinal (24 cases), axillary (one case), and mediastinal (five cases), may also be involved. However, no patient had ILNR in RSCLN. To the best of our knowledge, only one case report about metastatic OC of RSCLN has been published [11]. In that report, the patient had metastatic RSCLN involvement where the primary OC manifested after three years of clinical surveillance.

We hypothesize that the ILNR routes in OC include either lymphatic dissemination or lymphatic dissemination after transcoelomic spread. LSCLN collects lymph through the thoracic duct and from most areas of the body. In contrast, RSCLN takes its supply mainly from the mediastinum, lungs, and esophagus [1]; in a patient with OC, the route of ILNR in LSCLN is lymphatic dissemination, and that in RSCLN is lymphatic dissemination from the transcoelomic spread site. In the present case, although the diagnosis of ILNR was surgically confirmed, the suspected presence of a peritoneal lesion was based on PET/CT findings. Bristow et al. [12] documented the presence of occult intraperitoneal disease in 21.4% of ILNR cases even when PET/CT scanning techniques were used. Thus, we think that, in the present case, the route was possibly lymphatic dissemination from occult peritoneal disease.

Treatment for ILNR of OC is based on various factors, such as the recurrence site, the general condition of the patient, disease-free interval, growth rate, and response to chemotherapy. Although these confounding factors could be more adequately assessed through a multivariate analysis of a larger number of patients, there are several reports about therapy for ILNR of OC. Those reports indicated that surgical removal of ILNR with or without adjuvant therapy, such as chemotherapy, radiation therapy, or both, is associated with a favorable clinical outcome for a selected subgroup of patients with ILNR of OC [3,6-9]. In the present case, the patient underwent surgical resection of ILNR and adjuvant chemotherapy and was free of disease at a follow-up consultation 29 months after the first notice of relapse.

## Conclusions

Peripheral ILNR, including RSCLN, could occur without a macroscopic abdominal lesion. Clinicians should carefully examine peripheral lymph nodes for recurrence.

## Consent

Written informed consent was obtained from the patient for publication of this case report and any accompanying images. A copy of the written consent is available for review by the Editor-in-Chief of this journal.

## Abbreviations

RSCLN, Right supraclavicular lymph node; OC, Ovarian cancer; LSCLN, Left supraclavicular lymph node; CA125, Cancer-related antigen 125; CA19-9, Carbohydrate antigen 19–9; TC, Paclitaxel and carboplatin; AUC, Area under the curve; CT, Computed tomography; wTC, Weekly TC; PET/CT, Fluorine-18 fluorodeoxyglucose positron emission tomography associated with computed tomography; CK, Cytokeratin; WT1, Wilms’ tumor 1 protein; ILNR, Isolated lymph node recurrence.

## Competing interests

The authors declare that they have no competing interests.

## Authors’ contributions

TT participated in patient treatment, histology-related issues, and literature review, and drafted the manuscript. MO contributed to patient treatment and revised the corresponding sections in the manuscript. Both authors read and approved the final manuscript.

## References

[B1] Mizutani M Nawata S Hirai I Murakami G Kimura W Anatomy and histology of Virchow's node Anat Sci Int 2005 80 193 198 10.1111/j.1447-073X.2005.00114.x 16333915

[B2] Berek JS Berek JS, Hacker NF Epithelial ovarian cancer Practical Gynecologic Oncology 1994 3 Philadelphia: Lippincott Wilkins 466 467

[B3] Legge F Petrillo M Adamo V Pisconti S Scambia G Ferrandina G Epithelial ovarian cancer relapsing as isolated lymph node disease: natural history and clinical outcome BMC Cancer 2008 8 367 10.1186/1471-2407-8-367 19077269 PMC2632673

[B4] Blanchard P Plantade A Pagès C Afchain P Louvet C Tournigand C de Gramont A Isolated lymph node relapse of epithelial ovarian carcinoma: outcomes and prognostic factors Gynecol Oncol 2007 104 41 45 10.1016/j.ygyno.2006.06.039 16952391

[B5] Benedetti Panici P Perniola G Angioli R Zullo MA Manci N Palaia I Bellati F Plotti F Calcagno M Basile S Bulky lymph node resection in patients with recurrent epithelial ovarian cancer: impact of surgery Int J Gynecol Cancer 2007 17 1245 1251 10.1111/j.1525-1438.2007.00929.x 17425680

[B6] Santillan A Karam AK Li AJ Giuntoli R Gardner GJ Cass I Karlan BY Bristow RE Secondary cytoreductive surgery for isolated nodal recurrence in patients with epithelial ovarian cancer Gynecol Oncol 2007 104 686 690 10.1016/j.ygyno.2006.10.020 17141302

[B7] Uzan C Morice P Rey A Pautier P Camatte S Lhommé C Haie-Meder C Duvillard P Castaigne D Outcomes after combined therapy including surgical resection in patients with epithelial ovarian cancer recurrence(s) exclusively in lymph nodes Ann Surg Oncol 2004 11 658 664 10.1245/ASO.2004.11.023 15197013

[B8] Fotiou S Aliki T Petros Z Ioanna S Konstantinos V Vasiliki M George C Secondary cytoreductive surgery in patients presenting with isolated nodal recurrence of epithelial ovarian cancer Gynecol Oncol 2009 114 178 182 10.1016/j.ygyno.2009.04.025 19450872

[B9] Gadducci A Cosio S Zola P Sostegni B Ferrero AM Teti G Cristofani R Sartori E The clinical outcome of epithelial ovarian cancer patients with apparently isolated lymph node recurrence: a multicenter retrospective Italian study Gynecol Oncol 2010 116 358 363 10.1016/j.ygyno.2009.11.008 19954826

[B10] Zannoni GF Vellone VG Distefano MG Fadda G Scambia G Ovarian serous carcinoma presenting with mediastinal lymphadenopathy 20 months before the intraabdominal mass: role of immunohistochemistry Gynecol Oncol 2007 104 497 500 10.1016/j.ygyno.2006.09.030 17126890

[B11] Mayadevi S Nagarajan S Van Der Voet JC Nevin J Cruickshank DJ Metastatic adenocarcinoma of right supraclavicular fossa–delayed presentation of ovarian primary J Obstet Gynaecol 2005 25 528 529 10.1080/01443610500211627 16261694

[B12] Bristow RE Giuntoli RL Pannu HK Schulick RD Fishman EK Wahl RL Combined PET/CT for detecting recurrent ovarian cancer limited to retroperitoneal lymph nodes Gynecol Oncol 2005 99 294 300 10.1016/j.ygyno.2005.06.019 16051330

